# Relevance of proliferative and pro-apoptotic factors in non-small-cell lung cancer for patient survival

**DOI:** 10.1054/bjoc.1999.1210

**Published:** 2000-04-27

**Authors:** M Volm, R Koomägi

**Affiliations:** Department of Oncological Diagnostics and Therapy, German Cancer Research Center, Im Neuenheimer Feld 280, Heidelberg, D-69120, Germany

**Keywords:** non-small-cell lung carcinomas, proliferation, apoptosis, prognosis, survival

## Abstract

This investigation first set out to analyse which cellular proliferative and apoptotic factors, in addition to the clinical prognostic factors, are most predictive in patients with non-small-cell lung carcinomas (NSCLC). To this purpose, we related the proliferative factors proliferating cell nuclear antigen (PCNA), cyclin A, cyclin D1, cyclin-dependent kinase 2 (cdk2), cdk4 and the proportion of cell cycle phases in NSCLC to the survival times of 150 patients. Additionally, we associated the expressions of Fas, Fas ligand and caspase-3 in NSCLC to patient survival. Immunohistochemistry was used to determine the proteins and flow cytometry to assess the proportion of cell cycle phases. Patients with PCNA-positive carcinomas had significantly shorter survival times than patients with PCNA-negative carcinomas (median survival times: 51 vs 89 weeks). Corresponding results were obtained with the factor cyclin A (64 vs 92 weeks), with the factor cdk2 (76 vs 89 weeks), with the factor cdk4 (62 vs 102 weeks) and with the proportion of S phases (86 vs 121 weeks). Patients with an expression of the apoptotic factors had a more favourable prognosis than patients with negative carcinomas. The median survival times of cancer patients with Fas expression was 86 weeks and of those without Fas expression only 69 weeks. Corresponding results were obtained with the Fas ligand (87 vs 41 weeks) and caspase 3 (87 vs 34 weeks). In order to determine whether a combination of factors can yield improved prognostic information, we investigated all possible combinations of the proliferative and apoptotic factors. Patients with tumours having a high proliferative activity, but which did not express apoptotic factors had the shortest survival times while patients with a low proliferative activity and a high expression of apoptotic factors had the most favourable outcome. A multivariate analysis (Cox model) of the cellular and clinical prognostic factors indicated that stage, lymph node involvement, Fas, PCNA and cyclin A are the most important prognostic factors for the clinical outcome of patients with non-small-cell lung carcinomas. © 2000 Cancer Research Campaign


*British Journal of Cancer* (2000) **82**(10), 1747–1754
© 2000 Cancer Research Campaign
DOI: 10.1054/ bjoc.1999.1210, available online at http://www.idealibrary.com on IDEAL®

### Relevance of proliferative and pro-apoptotic factors in non-small-cell lung cancer for patient survival

**M Volm and R Koomägi**

Department of Oncological Diagnostics and Therapy, German Cancer Research Center, Im Neuenheimer Feld 280, D-69120 Heidelberg, Germany

**Summary** This investigation first set out to analyse which cellular proliferative and apoptotic factors, in addition to the clinical prognostic factors, are most predictive in patients with non-small-cell lung carcinomas (NSCLC). To this purpose, we related the proliferative factors proliferating cell nuclear antigen (PCNA), cyclin A, cyclin D1, cyclin-dependent kinase 2 (cdk2), cdk4 and the proportion of cell cycle phases in NSCLC to the survival times of 150 patients. Additionally, we associated the expressions of Fas, Fas ligand and caspase-3 in NSCLC to patient survival. Immunohistochemistry was used to determine the proteins and flow cytometry to assess the proportion of cell cycle phases. Patients with PCNA-positive carcinomas had significantly shorter survival times than patients with PCNA-negative carcinomas (median survival times: 51 vs 89 weeks). Corresponding results were obtained with the factor cyclin A (64 vs 92 weeks), with the factor cdk2 (76 vs 89 weeks), with the factor cdk4 (62 vs 102 weeks) and with the proportion of S phases (86 vs 121 weeks). Patients with an expression of the apoptotic factors had a more favourable prognosis than patients with negative carcinomas. The median survival times of cancer patients with Fas expression was 86 weeks and of those without Fas expression only 69 weeks. Corresponding results were obtained with the Fas ligand (87 vs 41 weeks) and caspase 3 (87 vs 34 weeks). In order to determine whether a combination of factors can yield improved prognostic information, we investigated all possible combinations of the proliferative and apoptotic factors. Patients with tumours having a high proliferative activity, but which did not express apoptotic factors had the shortest survival times while patients with a low proliferative activity and a high expression of apoptotic factors had the most favourable outcome. A multivariate analysis (Cox model) of the cellular and clinical prognostic factors indicated that stage, lymph node involvement, Fas, PCNA and cyclin A are the most important prognostic factors for the clinical outcome of patients with non-small-cell lung carcinomas. © 2000 Cancer Research Campaign

**Keywords**: non-small-cell lung carcinomas; proliferation; apoptosis; prognosis; survival

The majority of bronchogenic carcinomas can be histologically classified into four types: small-cell lung carcinomas, adenocarcinomas, squamous cell lung carcinomas and large-cell carcinomas. The histological features, clinical course and response to therapy indicate that small-cell lung carcinomas are a separate entity. The behaviour of the other three histological subtypes is similar. Therefore, these are combined within the larger group of non-small-cell lung carcinomas (NSCLCs). NSCLCs represent 75% of all cases of lung cancer and are usually associated with a poor prognosis. The present investigation is limited to NSCLC. In addition to pertinent clinical data, new risk factors at the molecular and cellular level are the subject of many ongoing studies. Predictive and prognostic factors can serve many purposes. They are used to understand the natural history of cancer, to identify homogeneous patient populations, to characterize subsets of patients with a potentially favourable or unfavourable outcome, to predict the success of therapy or to generate follow-up strategies.

It is a characteristic of all tumours to grow. However, tumours are complex cell populations in which cell gain and loss occur concurrently. In this investigation we analysed the relationship between patient survival and the proliferative activity and apoptosis in 150 NSCLCs in order to ascertain the implications of these factors for the prognosis of the patients.

*Received 5 October 1999*
*Revised 17 December 1999*
*Accepted 20 December 1999*

*Correspondence to:* M Volm

A variety of cellular changes can be used to evaluate cell proliferation in tumour material. Cyclins and cyclin-dependent kinases (cdks) are universal regulators of cellular progression in eukaryotic cells (Lew and Kornbluth, 1996). Regulatory mechanisms include variations in cyclin abundance, phosphorylation of the kinase subunit that may yield either positive or negative effects and the actions of cyclin-kinase inhibitory proteins. Several classes of mammalian cyclins that are synthesized in various ways and degraded at specific points during the cell cycle have been described (Cordon-Cardo, 1995). The complex formed by cyclin D1 and cdk4 governs the G1 progression, while cyclin A together with cdk2 regulates entry into and progression through the S phase. Another marker is the proliferating cell nuclear antigen (PCNA) which is essential for cellular DNA synthesis. (Jashulski et al, 1988; Garcia et al, 1989). In this study we examined the prognostic value of cyclin A, cyclin D, cdk2, cdk4 and PCNA through immunohistochemical analysis. Additionally we determined the cell cycle phases by using flow cytometry.

Apoptosis or programmed cell death is one of the most important regulatory mechanisms of cellular homeostasis in organisms. In this investigation we employed immunohistochemistry to determine the pro-apoptotic factors Fas/CD95, Fas ligand and caspase-3. Fas is a transmembrane protein (receptor) which, upon binding to its ligand (Fas ligand), transmits an intracellular signal that leads to programmed cell death (Fisher, 1994; Nagata and

Golstein, 1995). As previously reported, Fas and Fas ligand are expressed in different tissues including lung cancer (Hellquist et al, 1997; Niehans et al, 1997; Xerri et al, 1997). Caspases are key effectors of cellular death. Of the caspases, caspase-3 (also known as CPP32, Yama or apopain) is probably the one that so far best correlates with apoptosis (Fernandez-Alnemri et al, 1994; Nicholson et al, 1995).

The main purpose of the current study was to initially discover the most important prognostic factors for NSCLCs and then to examine whether a combination of proliferative and apoptotic factors can result in improved prognostic information for overall patient survival.

### MATERIALS AND METHODS

### Patients

One hundred and fifty patients with previously untreated NSCLC were admitted into this study. All patients (135 men, 15 women) underwent surgery in the Chest Hospital Heidelberg-Rohrbach. The morphological classification of the carcinomas was conducted according to World Health Organization (WHO) specifications. Of the carcinomas, 85 (56.6%) were squamous carcinomas, 40 (26.7%) were adenocarcinomas and 25 (16.7%) were large-cell carcinomas. All patients were staged at the time of their surgery according to the guidelines of the American Joint Committee on Cancer. Thirty-two patients had stage I, 11 stage II and 107 patients had stage IIIA tumours. The average age of the patients was 58 (range 25–76) years. Sixty-three patients (42%) did not exhibit lymph node involvement while 87 did (58%). One hundred and nine patients were given only surgical treatment. Fifteen patients were also given cytotoxic drugs and 25 patients (mainly squamous cell lung carcinomas) were treated with palliative irradiation. The additional radiation treatment and chemotherapy had no significant effect on overall patient survival time ($P > 0.1$). Follow-up data were obtained from hospital charts and by corresponding with the referring physicians.

### Antibodies

To detect the proliferative activity, anti-cyclin A (clone H-432, dilution 1:50), anti-cdk2 (clone M2, dilution 1:200) and anti-cdk4 (clone C-22, dilution 1:100) were used. These were obtained from Santa Cruz Biotechnology (Santa Cruz, CA, USA). Anti-cyclin D1 (clone Ab-3, dilution 1:10) was produced by Calbiochem/Novabiochem (Baden-Soden, Germany) and the PCNA (clone PC10, dilution 1:10) was obtained from Dianova (Hamburg, Germany). The antibody to detect the apoptotic factor Fas (clone UB2, dilution 1:100) originated from Immunotech (Hamburg, Germany) and the antibodies to detect the apoptotic factors Fas ligand (clone Q20, dilution 1:500) and caspase-3 (clone CPP32 p20 [E-8], dilution 1:500) were products of Santa Cruz Biotechnology.

### Immunohistochemistry

The previously described biotin–streptavidin method was used to detect the proteins (Volm et al, 1993, 1997b; Koomägi and Volm, 1999). Formalin-fixed and paraffin-embedded tissue were deparaffinized and subsequently microwaved in citrate buffer. After preincubation with hydrogen peroxide, and protein blocking solution, the primary antibodies were applied for 16 h at 4°C. After incubation with secondary antibodies, the streptavidin biotinylated peroxidase complex was added and the peroxidase activity visualized with 3-amino-9-ethylcarbazole. Counter-staining was performed with haematoxylin. Negative and positive controls were performed. The specificity of the antibodies was confirmed by immunoblotting. Without having any prior knowledge of each patient's clinical data, three observers independently evaluated the results from the immunohistochemical staining. If the evaluations did not agree (< 10%) the specimens are reevaluated and then classified according to the assessment given most frequently by the observers. The immunohistochemical staining was analysed according to a scoring method that we have previously validated in a series of animal and human cell lines and human solid tumours. Finally, we classified the tumours into four groups: tumours without staining and tumours with weak, moderate and strong staining.

### Western blot analysis

Protein was isolated with the Tri-reagent (MRC, Cincinnati, OH, USA). After electrophoresis on a 12% polyacrylamide gel in the presence of sodium dodecyl sulphate (SDS) and transfer to a polyvinyl difluoride (PVDF) membrane (NEN, Boston, MA, USA) by electroblotting, the transferred protein and molecular weight markers were detected with 0.3% Ponceau S. Blocking in 1% blocking solution (Western Blocking Reagent, Boehringer Mannheim, Germany) preceded the 1-h-long incubation with the antibody diluted in 0.5% blocking solution. Thereafter, peroxidase-conjugated streptavidin secondary antibodies (Santa Cruz) were used to detect the proteins. All incubations were conducted at room temperature and several washing steps followed each incubation. Signals were detected with chemiluminescence or with 4-chloro-1-naphthol.

### Cell cycle analysis

A mixture of propidium iodide and 4′-6-diamidino-2-phenylindole was applied simultaneously with RNAase after methanol fixation and protease digestion of single cell suspensions (Haag, 1980). Flow cytometry analysis was undertaken with an ICP-22 (Phywe, Göttingen, Germany). Peripheral blood leucocytes from healthy donors were used as a calibration standard for DNA diploidy. Parallel measurements, both including and omitting the standard, were performed. The cell cycle analysis was performed using integrated Gaussian fittings. A computerized subtraction of exponentially decreasing corrections beginning with the peak of cellular debris was included in the evaluation program. The cell cycle analysis was omitted in cases that exhibited interspersed cell populations.

### Statistical analysis

Patient survival time was determined from the date of surgery until the last follow-up visit or reported death and was evaluated by using life table analyses according to the method of Kaplan and Meier. Groups were compared by using the log-rank test. The correlations between clinical and molecular parameters were statistically evaluated by using Fisher's exact test. This test was used as a statistical hypothesis test for the presence or absence of a relationship between two factors. A $P$-value of < 0.05 was

© 2000 Cancer Research Campaign

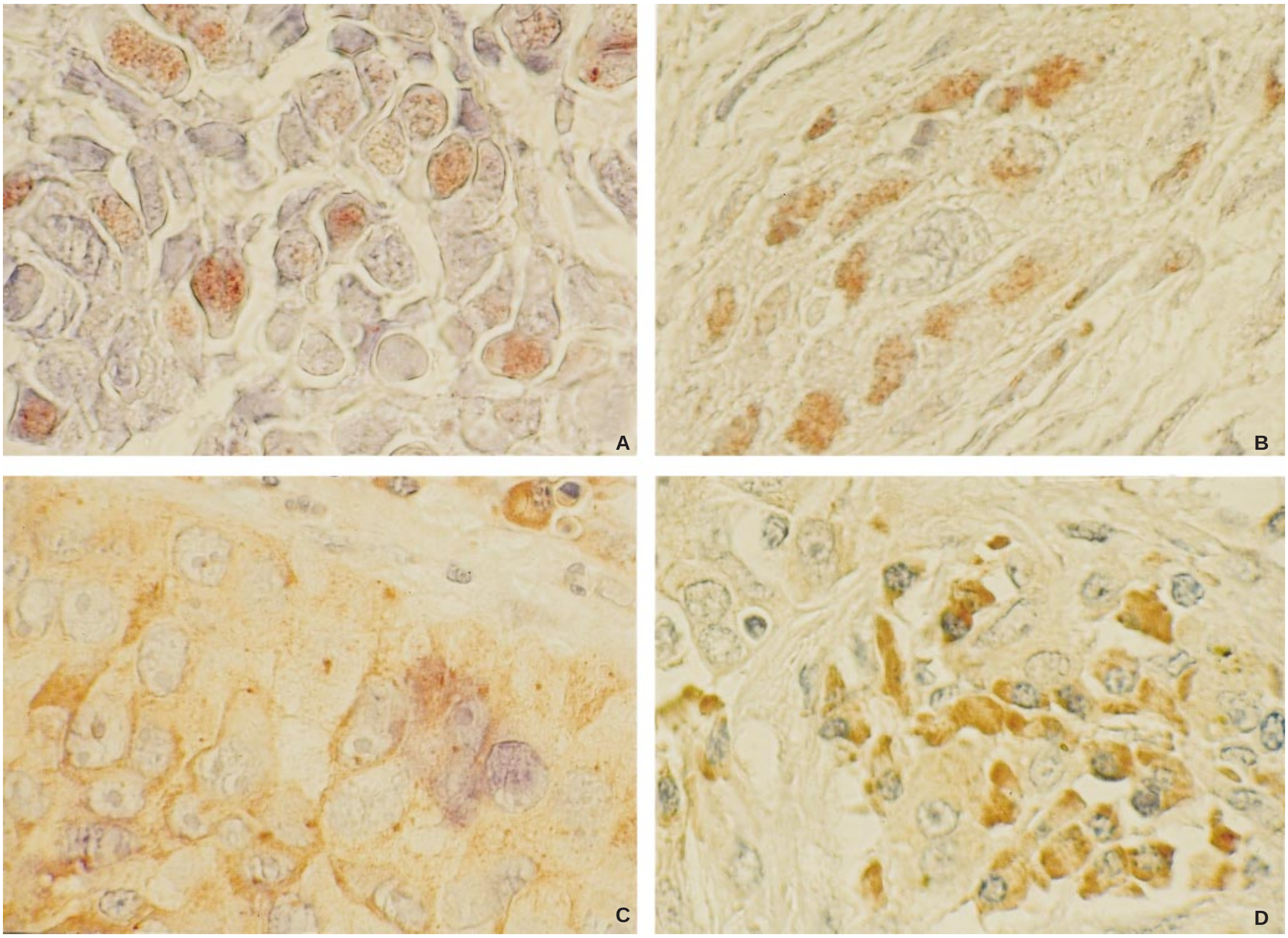

**Figure 1** Immunohistochemical staining of PCNA (**A**), cyclin A (**B**), Fas (**C**) and caspase-3 (**D**)

considered significant. For the analysis itself, tumours without staining and tumours with weak staining were classified as negative and tumours with moderate to strong staining were classified as positive.

### RESULTS

Immunohistochemistry was used to determine the proliferative factors PCNA, cyclin A, cyclin D1, cdk2, cdk4 and the pro-apoptotic factors Fas, Fas-ligand and caspase-3 in formalin-fixed, paraffin-embedded specimens from 150 NSCLCs of previously untreated patients. Figure 1 shows representative protein expression patterns of PCNA (Figure 1A), cyclin A (Figure 1B), Fas (Figure 1C) and caspase-3 (Figure 1D) which reveal nuclear (PCNA, cyclin A), membrane (Fas) or cytoplasmatic (caspase-3) immunoreactivity. The specificity of the staining was confirmed by Western blots (Figure 2). DNA histograms representative of our experimental data are shown in Figure 3.

### Clinical prognostic factors

The overall survival of patients with NSCLCs is mainly determined by tumour extent, lymph node status and stage. This also applies to our patients (Figure 4 and Table 1).

### Cellular prognostic factors

In order to discover new prognostic factors at the cellular level, we determined the expression of different proliferative factors in NSCLCs. We then detected a relationship between patient survival and the proliferative factors. Patients with PCNA-positive carcinomas had significantly shorter survival times than patients with PCNA-negative carcinomas (51 vs 89 weeks, Table 2). The relative risk for patients with PCNA-positive carcinomas was 1.5 compared to patients with PCNA-negative carcinomas. Corresponding results were obtained with the factors cyclin A, with the factor cdk2, and with the factor cdk4 (Table 2). To confirm these results, we used flow cytometry to analyse the S phase proportion of the lung carcinomas. Consistent with the data obtained by immunohistochemistry, we found that the survival times were shorter for cancer patients with a high S phase proportion than for patients with low S phase tumours (Table 2). In contrast, no correlation was found between the expression of cyclin D1 and a patient's outcome.

Apoptosis is regulated by a variety of pro-apoptotic and anti-apoptotic factors. In this study, we measured the pro-apoptotic factors Fas, Fas ligand and caspase-3 and discovered an inverse relationship between the apoptotic factors and patient survival. Patients with a greater expression of the apoptotic factors had a

© 2000 Cancer Research Campaign

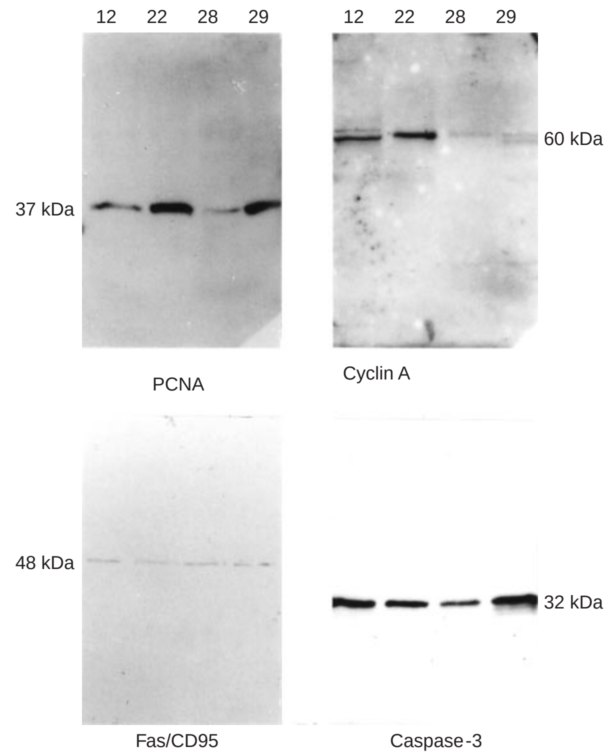

**Figure 2** Immunoblot analyses (Western blots) of the carcinomas Nos. 12, 22, 28, 29. Detection of PCNA, cyclin A, Fas and caspase-3. PCNA, cyclin A and caspase-3 were detected by chemoluminescence; Fas was detected by 4-chloro-1-naphthol.

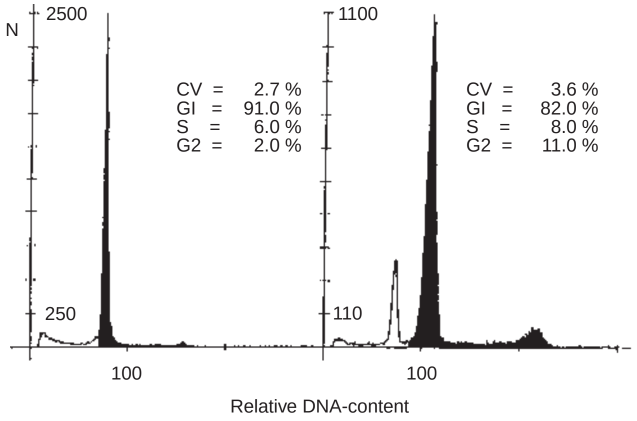

**Figure 3** Representative examples of the DNA-histograms of lung carcinomas. Cell cycle analysis was performed in areas marked by darkening. CV = coefficient of variation more favourable prognosis. The median survival times of cancer patients with Fas expression was 86 weeks and of those without Fas expression only 69 weeks (Table 3). The relative risk estimate for patients with Fas-negative carcinomas was 1.5 compared to patients with Fas-positive carcinomas. Corresponding results were obtained with the Fas ligand. Additionally, we analysed the relationship between caspase-3 and patient survival, because caspases are key effectors of cellular death and, of the various caspases, caspase-3 is the one that so far best correlates with apoptosis. Analogous to the findings with Fas and Fas ligand, we determined

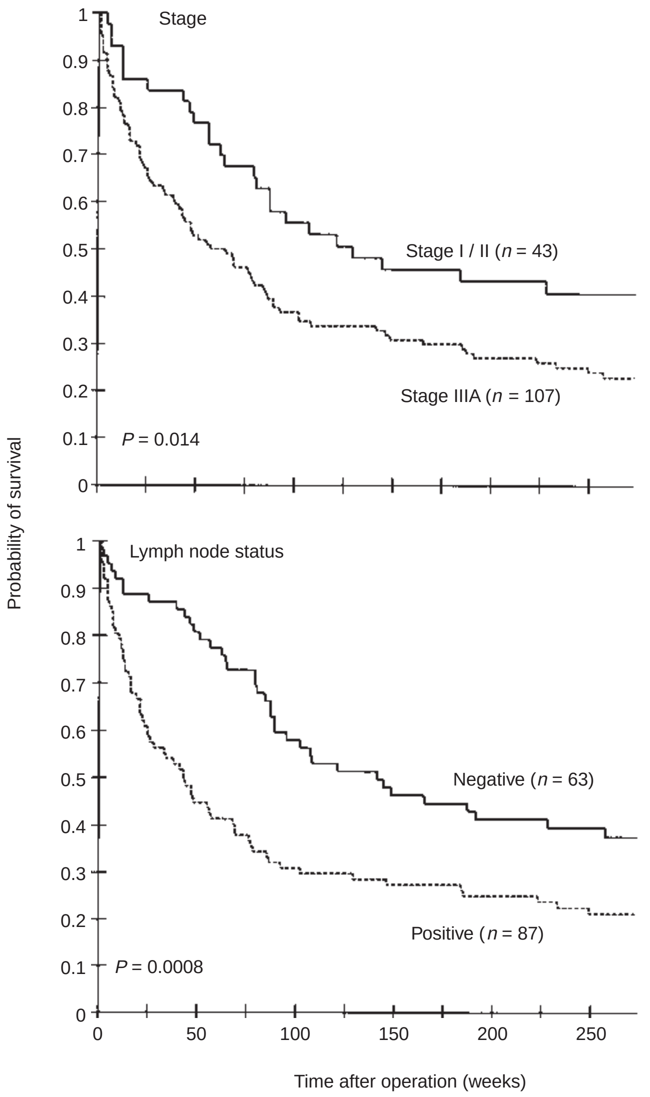

**Figure 4** Survival curves (Kaplan–Meier estimates) of patients with NSCLC grouped according to stage (above) and lymph node involvement (below)

that patients who expressed caspase-3 had a favourable outcome (Table 3).

### Combinations of factors

An analysis of the inter-relationships of the cellular factors indicated that a correlation does not exist among the proliferative and the apoptotic factors (data not shown). In order to determine whether a combination of factors can yield improved prognostic information, we investigated all possible combinations of the proliferative and apoptotic factors. Figures 5 to 8 show the results of the combinations of the different proliferative factors and the pro-apoptotic factor Fas. Patients with tumours having a high proliferative activity, but which did not express Fas had the shortest survival times while patients with a low proliferative activity and a high expression of Fas had the most favourable outcome. In Table 4 the median survival times and the relative risks of patients grouped according to the proliferative activity and

 *© 2000 Cancer Research Campaign*

**Table 1** Median survival times (MST) and relative risk of patients with non-small cell lung carcinomas according to clinical variables (*n* = 150)

| Clinical variables | Patients/ death | MST (weeks) | Log-rank Test *P*-value | Relative risk |
|---|---|---|---|---|
| Tumour extent | | | | |
| T1,2 | 56/34 | 107 | | 1.0 |
| T3 | 94/73 | 65 | 0.021 | 1.6 |
| LN involvement | | | | |
| Negative | 63/38 | 141 | | 1.0 |
| Positive | 87/69 | 43 | 0.0008 | 1.9 |
| Stage | | | | |
| I, II | 43/25 | 129 | | 1.0 |
| IIIA | 107/82 | 65 | 0.014 | 1.7 |

**Table 2** Median survival times (MST) and relative risk of patients with NSCLCs according to proliferative factors

| Proliferative factors | Patients/ death | MST (weeks) | Log-rank test *P*-value | Relative risk |
|---|---|---|---|---|
| PCNA | | | | |
| Negative | 99/67 | 89 | | 1.0 |
| Positive | 43/34 | 51 | 0.049 | 1.5 |
| Cyclin A | | | | |
| Negative | 65/41 | 92 | | 1.0 |
| Positive | 75/58 | 64 | 0.089 | 1.4 |
| Cyclin D1 | | | | |
| Negative | 60/42 | 77 | | 1.0 |
| Positive | 85/61 | 84 | 0.864 | 1.0 |
| cdk2 | | | | |
| Negative | 39/26 | 89 | | 1.0 |
| Positive | 105/76 | 76 | 0.362 | 1.2 |
| cdk4 | | | | |
| Negative | 80/53 | 102 | | 1.0 |
| Positive | 59/44 | 62 | 0.135 | 1.4 |
| S phases | | | | |
| < 9.5% | 32/19 | 121 | | 1.0 |
| > 9.5% | 39/29 | 86 | 0.147 | 1.5 |

Tumour material was not available for all measurements. The cell cycle analysis was omitted in cases that exhibited interspersed cell populations.

**Table 3** Median survival times (MST) and relative risk of patients with NSCLCs according to apoptotic factors

| Apoptotic factors | Patients/ death | MST (weeks) | Log-rank test P-value | Relative risk |
|---|---|---|---|---|
| Fas/CD95 | | | | |
| Negative | 71/58 | 69 | | 1.5 |
| Positive | 78/48 | 86 | 0.022 | 1.0 |
| Fas ligand | | | | |
| Negative | 37/31 | 41 | | 1.6 |
| Positive | 90/62 | 87 | 0.049 | 1.0 |
| Caspase-3 | | | | |
| Negative | 26/20 | 34 | | 1.5 |
| Positive | 69/49 | 87 | 0.157 | 1.0 |

Tumour material was not available for all measurements.

the level of carcinoma Fas expression are listed. The median survival time of patients with PCNA-positive/Fas-negative carcinomas was only 19 weeks while the median survival time of patients with PCNA-negative/Fas-positive carcinomas was 146 weeks. The relative risk for the first group of patients was 2.6. Corresponding results were obtained with the other combinations

© 2000 Cancer Research Campaign

**Table 4** Median survival times (MST) and relative risk of patients with NSCLCs according to proliferative and apoptotic factors

| Combinations | Patients/ death | MST (weeks) | Log-rank test *P*-value | Relative risk |
|---|---|---|---|---|
| PCNA-pos/Fas-neg | 18/16 | 19 | | 2.6 |
| PCNA-neg/Fas-pos | 49/27 | 146 | 0.002 | 1.0 |
| Cyclin A-pos/Fas-neg | 31/28 | 77 | | 2.7 |
| Cyclin A-neg/Fas-pos | 31/15 | > 260 | 0.001 | 1.0 |
| Cdk4-pos/Fas-neg | 28/23 | 38 | | 2.0 |
| Cdk4-neg/Fas-pos | 42/23 | 184 | 0.016 | 1.0 |
| S phase high/Fas neg | 18/16 | 38 | | 3.0 |
| S phase low/Fas-pos | 18/8 | > 260 | 0.007 | 1.0 |

**Table 5** Multivariate analyses of the prognostic value of stage or lymph node involvement and the cellular factors Fas, PCNA and cyclin A

| Variables | *P*-values | Variables | *P*-values |
|---|---|---|---|
| Stage | 0.009 | LN | 0.001 |
| Fas | 0.007 | Fas | 0.026 |
| PCNA | 0.042 | PCNA | 0.040 |
| Stage | 0.016 | LN | 0.003 |
| Fas | 0.0008 | Fas | 0.033 |
| Cyclin A | 0.097 | Cyclin A | 0.046 |

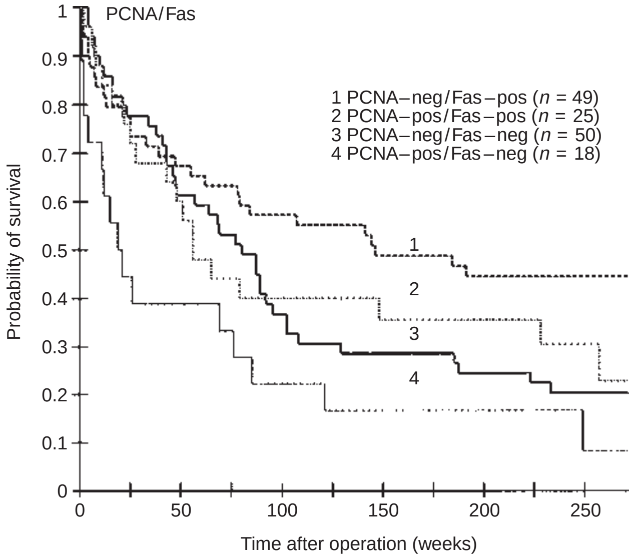

**Figure 5** Survival times of patients (Kaplan–Meier estimates) with NSCLCs according to the expression of a proliferative (PCNA) and an apoptotic factor (Fas)

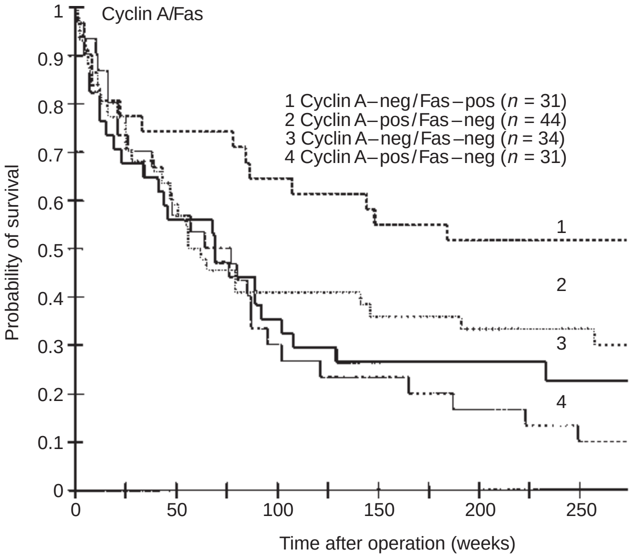

**Figure 6** Survival times of patients (Kaplan–Meier estimates) with NSCLCs according to the expression of a proliferative (cyclin A) and an apoptotic factor (Fas)

(Table 4). Similar results were obtained with the proliferative factors and the apoptotic factors Fas ligand and caspase-3 (data not shown).

A multivariate analysis (Cox model) of the cellular and clinical prognostic factors indicated that stage, lymph node involvement, Fas, PCNA and cyclin A are the most important prognostic factors for the clinical outcome of patients with NSCLCs. Since a very high correlation exists between stage and lymph node involvement and between PCNA and cyclin A, these factors have been considered separately (Table 5). Along with stage, the pair Fas and PCNA gives the best prognostic separation. Along with lymph node involvement, the pairs Fas/PCNA and Fas/cyclin A give the best prognostic information.

### DISCUSSION

In addition to established factors such as the extent of a tumour, lymph node involvement and the particular stage of the disease in lung carcinomas, the search for new risk factors is an ongoing undertaking. The balance between proliferation and apoptosis

 *© 2000 Cancer Research Campaign*

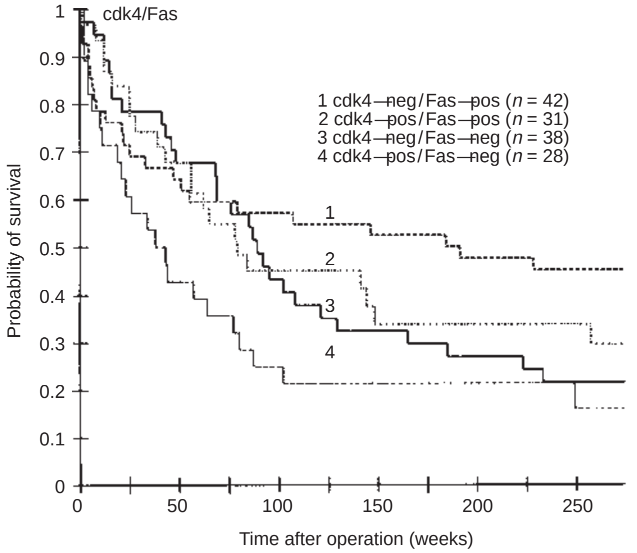

**Figure 7** Survival times of patients (Kaplan–Meier estimates) with NSCLCs according to the expression of a proliferative (cdk4) and an apoptotic factor (Fas)

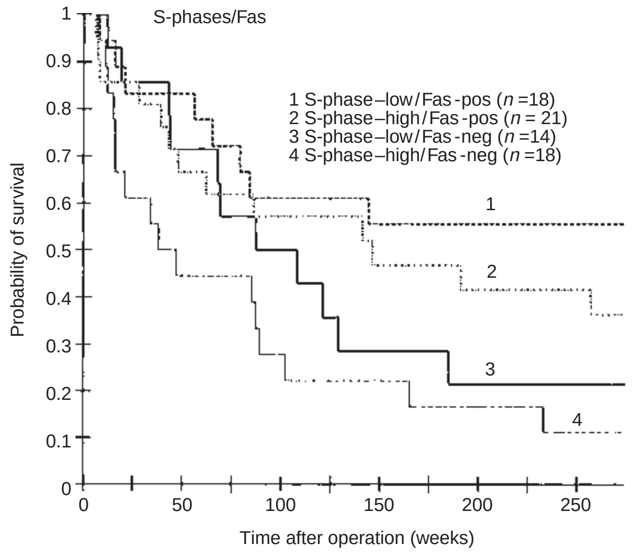

**Figure 8** Survival times of patients (Kaplan–Meier estimates) with NSCLCs according to the expression of a proliferative (S phases) and an apoptotic factor (Fas)

within a tissue is important in controlling its overall growth and these factors may, therefore, also prove useful in an assessment of a patient's prognosis.

Information related to cell kinetics can be a useful adjunct in understanding the behaviour of tumours. In a variety of malignant neoplasms, correlations have been noted between proliferation, recurrence and overall survival (Hall et al, 1990). Different methods have been used to study cell kinetics in tumour samples. Methods for assessing the proliferative activity in vitro or in vivo required labelling with [$^3$H]-thymidine, cytometry to examine the relative cellular DNA content and the application of immunohistochemical techniques. The current study determined the proliferative activity of the carcinomas with immunohistochemistry by using antibodies against the proteins PCNA, cyclin A, cyclin D, cdk2, cdk4 and by measuring the cell cycle with flow cytometry.

Experiments using antisense oligonucleotides suggest that the gene coding for PCNA is essential for cellular DNA synthesis (Jaskulski et al, 1988). In the present investigation, we found that patients who had lung tumours with a high proportion of PCNA-positive cells exhibited shorter median survival times (51 weeks) than did lung tumour patients with a low proportion of PCNA-positive cells (89 weeks). Our finding agrees with the data reported by Ebina et al (1994). Cyclin A has also been reported to be involved in proliferation (Zindy et al, 1992). Along with other researchers, we could demonstrate that cyclin A expression closely correlates with the proportion of S phase cells as measured by flow cytometry (Paterlini, 1995; Volm et al, 1997a). The current study shows that patients with cyclin A-positive carcinomas have shorter survival times than those with cyclin A-negative carcinomas. Following mitogenic stimulation, cells in the late G1 phase exhibit a rapid increase in the level of cyclin D (Tam et al, 1994). Cyclin D1 is overexpressed in several types of tumours, because of gene amplification or chromosomal rearrangement (Keyomarsi and Pardee, 1993). However, the reported data on the prognostic value of cyclin D1 are inconsistent (Michalides et al, 1995; Naitoh et al, 1995; Sheshadri et al, 1996). In an earlier study (Volm et al, 1996), we found that the take rate of human squamous cell lung carcinomas in nude mice was significantly higher when the carcinomas expressed cyclin D1. In the present investigation, we did not observe a relationship between the expression of cyclin D1 and patient outcome. It is postulated that the complexes formed by cyclin D1 and cdk4 govern G1 progression and that cyclin A/cdk2 regulates progression through the S phase (Cordon-Cardo, 1995). In our study, patients with cdk2-positive and cdk4-positive carcinomas had shorter overall survival times, but the differences between cdk-positive and cdk-negative groups were statistically insignificant. In short, we found a relationship between patient survival and the investigated proliferative factors as measured by immunohistochemistry. These results were confirmed by determining the S phases with flow cytometry.

Tumours remain dormant when tumour cell proliferation is counterbalanced by an equivalent rate of cell death. The Fas receptor/Fas ligand system and caspase-3 are primarily responsible for the induction of apoptosis. To analyse the relationship between the clinical outcome and apoptosis, the expression of these three factors was, therefore, analysed by immunohistochemistry. Our results show that the survival times were longer both in patients with Fas-positive and Fas ligand-positive carcinomas. This is also true for patients with caspase-3-positive carcinomas. Kangas et al (1998) found that caspase-3 plays an important role in c-Myc-induced apoptosis. Caspases are key effectors of cellular death and, of the various caspases, caspase-3 is the one that so far best correlates with apoptosis (Nicholson et al 1995). We could not find a correlation between apoptotic index (TUNEL-assay) and the survival time of patients (data not shown). This can be explained with data reported by Kangas et al (1998). Apoptotic cells are rapidly phagocytized and therefore, the evaluation of the apoptotic index is limited and may underestimate the number of apoptotic cells. Further, the TUNEL-assay can only detect apoptotic cells at a late stage, therefore, the relationships of the expression of pro-apoptotic factors and apoptotic indices that are detected at an earlier stage are rather weak.

In order to determine whether a combination of the proliferative and apoptotic factors may result in improved prognostic information,

© 2000 Cancer Research Campaign we examined all possible combinations of the investigated factors. This systematic undertaking which also assessed stage and lymph node involvement indicated that stage, lymph node status, Fas, PCNA and cyclin A are the most important prognostic factors for the clinical outcome of patients with non-small cell lung carcinomas. The pair FAS and PCNA yield the best prognostic separation along with stage or lymph node status. Proliferation and apoptosis are under complex molecular control and both can be triggered by diverse signals. By limiting the supply of such signals, tight control can be exerted over cell numbers. If the rate of cell death exceeds the proliferation rate, tumour regression will occur. Conversely, if proliferation exceeds cell death, tumour progression will result. Our results indicate that both cellular proliferation and cell loss must be determined and that an evaluation of the proliferation to apoptosis ratio may very well be more important than the isolated assessment of either.

### ACKNOWLEDGEMENTS

The authors wish to thank Professor I Vogt-Moykopf and Professor P Drings of the Chest Hospital Heidelberg-Rohrbach for providing surgical material used in this study. We would also like to thank J Boldrin and A Wittmann for their excellent technical assistance.

### REFERENCES

Cardon-Cardo C (1995) Mutation of cell cycle regulators. Biological and clinical implications for human neoplasia (review). *Am J Pathol* **147**: 545–560

Ebina M, Steinberg SM, Mulshine JL and Linnoila RI (1994) Relationship of p53 overexpression and up-regulation of proliferating cell nuclear antigen with the clinical course of non-small-cell lung cancer. *Cancer Res* **54**: 2496–2503

Fernandez-Alnemri T, Litwack G and Alnemri ES (1994) CPP32, a novel human apoptotic protein with homology to *Caenorhabditis elegans* cell death Ced-3 and mammalian interleukin-1β-converting enzyme. *J Biol Chem* **269**: 30761–30764

Fisher DE (1994) Apoptosis in cancer therapy: crossing the threshold. *Cell* **78**: 539–542

Garcia RL, Coltera MD and Gown AM (1989) Analysis of proliferative grade using anti-PCNA/cyclin monoclonal antibodies in fixed, embedded tissues. *Am J Pathol* **134**: 733–773

Haag D (1980) Flow microfluorometric deoxyribonucleic acid (DNA) analysis supplementing routine histopathologic diagnosis of biopsy specimens. *Lab Invest* **42**: 85–90

Hall PA and Levison DA (1990) Review: assessment of cell proliferation in histological material. *J Clin Pathol* **43**: 184–192

Hellquist HB, Alejnick B, Jadner M, Andersson T and Sederholm C (1997) Fas receptor is expressed in human lung squamous cell carcinomas whereas bcl-2 and apoptosis are not pronounced. A preliminary report. *Br J Cancer* **76**: 175–179

Jaskulski D, De Riel JK, Mercer WE, Calabretta B and Baserga R (1988) Inhibition of cellular proliferation by antisense oligodeoxynucleotides to PCNA/cyclin. *Science* **240**: 1544–1546

Kangas A, Nicholson DW and Hölttä E (1998) Involvement of CPP32/caspase-3 in c-Myc-induced apoptosis. *Oncogene* **16**: 387–398

Keyomarsi K and Pardee AB (1997) Redundant cyclin over-expression and gene amplification in breast cancer cells. *Proc Natl Acad Sci USA* **90**: 1112–1116

Koomägi R and Volm M (1999) Expression of Fas (CD95/Apo-1) and Fas ligand in lung cancer, its prognostic and predictive relevance. *Int J Cancer (Pred Oncol)* **84**: 239–243

Lew DJ and Kornbluth S (1996) Regulatory roles of cyclin-dependent kinase phosphorylation in cell cycle control. *Curr Opin Cell Biol* **8**: 795–804

Michalides R, Van Veelen N, Hart A, Loftus B, Wientjens E and Balm A (1995) Overexpression of cyclin D1 correlates with recurrence in a group of forty-seven operable squamous-cell carcinomas of the head and neck. *Cancer Res* **55**: 975–978

Nagata S and Golstein P (1995) The Fas death factor. *Science* **267**: 1451–1455

Naitoh H, Shibata J, Kawaguchi A, Kodama M and Hattori T (1995) Overexpression and localization of cyclin D1 RNA and antigen in esophageal cancer. *Am J Pathol* **146**: 1161–1169

Nicholson DW, Ali A, Thornberry NA, Vaillancourt JP, Ding CK, Gallant M, Gareau Y, Griffin PR, Labelle M and Lazebnik YA (1995) Identification and inhibition of the ICE/CED-3 protease necessary for mammalian apoptosis. *Nature* **376**: 37–43

Niehans GA, Brunner T, Frizelle SP, Liston JC, Salerno CT, Knapp DJ, Green DR and Kratzke RA (1997) Human lung carcinomas express Fas ligand. *Cancer Res* **57**: 1007–1012

Paterlini P, Flejou JF, DeMitri MS, Pisi E, Franco D and Brechot C (1995) Structure and expression of the cyclin A gene in human primary liver cancer. Correlation with flow cytometry parameters. *J Hepatol* **23**: 47–52

Seshadri R, Lee CSL, Hui R, McCaul K, Horsfall DJ and Sutherland RL (1996) Cyclin D-1 amplification is not associated with reduced overall survival in primary breast cancer, but may predict early relapse in patients with features of good prognosis. *Clin Cancer Res* **2**: 1177–1184

Tam SW, Theodoras AM, Shay JW, Draetta GF and Pagano M (1994) Differential expression and regulation of cyclin-D1 protein in normal and tumor human cells: association with cdk4 is required for cyclin-D1 function in G1 progression. *Oncogene* **9**: 2663–2674

Volm M, Mattern J and Samsel B (1993) Relationship of inherent resistance to doxorubicin, proliferative activity and expression of P-glycoprotein and glutathione S-transferase-π in human lung cancer. *Cancer* **71**: 3181–3187

Volm M, Stammler G, Koomägi R and Mattern J (1996) Co-expression of cyclin D1 and retinoblastoma gene product (pRb) in human squamous cell lung carcinomas is associated with increased tumor take rate in nude mice. *Int J Oncol* **9**: 1253–1257

Volm M, Koomägi R, Mattern J and Stammler G (1997*a*) Cyclin A is associated with an unfavourable outcome in patients with non-small-cell lung carcinomas. *Br J Cancer* **75**: 1174–1778

Volm M, Mattern J, Stammler G, Royer-Pokora B, Schneider S, Weirich A and Ludwig R (1997*b*) Expression of resistance related proteins in nephroblastoma after chemotherapy. *Int J Cancer* **63**: 193–197

Xerri L, Devilard E, Hassoun J, Mawa SC and Birg F (1997) Fas ligand is not only expressed in immune privileged human organs but is also coexpressed with Fas in various epithelial tissues. *J Clin Pathol Mol Path* **50**: 87–91

Zindy F, Lamas E, Chenivesse X, Sobczak I, Wang I, Fesquet D, Henglein B and Brechot C (1992) Cyclin A is required in S-phase in normal epithelial cells. *Biochem Biophys Res Commun* **182**: 1144–1151

 *© 2000 Cancer Research Campaign*
